# Supplementary material for: Community myths and misconceptions about sexual health in Tanzania: Stakeholders’ views from a qualitative study in Dar es Salaam Tanzania
Source: PLoS One. 2023 Feb 10;18(2):e0264706. doi: 10.1371/journal.pone.0264706 (PMC9916544; doi:10.1371/journal.pone.0264706)
Supplement: S1 File — (DOCX) [file pone.0264706.s002.docx]

Sexual and Reproductive Health - SRH

Sexually transmissible infections - STIs

World Health Organization - WHO

Human immunodeficiency virus – HIV

Muhimbili University of Health and Allied Sciences - MUHAS

Pan American Health Organization – PAHO

Health Care Providers -HCP

Non-governmental organizations - NGO

Antiretroviral therapy - ARV
