# Supplementary material for: Community myths and misconceptions about sexual health in Tanzania: Stakeholders’ views from a qualitative study in Dar es Salaam Tanzania
Source: PLoS One. 2023 Feb 10;18(2):e0264706. doi: 10.1371/journal.pone.0264706 (PMC9916544; doi:10.1371/journal.pone.0264706)
Supplement: S2 File — (DOCX) [file pone.0264706.s003.docx]

**S1 Code book for sexual health myths and misconception.**

| Code | Description | Example/s |
| --- | --- | --- |
| Negatives perceptions on Family Planning and vaccines | The perceived impact to the use of family planning and vaccines such as HPV. | *-They think family planning is not of good intention. They say they (white people) want to decrease the size of the population in sub-Saharan Africa*  *-Some of African countries, you find parents are refusing to give consent and say that there is some hidden agenda behind the vaccine.* |
| Sex as a ritual | This involves engaging in a sexual act, either knowingly or unknowingly in a certain manner as a means of treating a certain disease or achieving something. | *traditional healer tells you must go have sex with him or a young girl or a young boy, to achieve something* |
| Mislabelling of HIV patients | This includes Internalized negative perception and stereotypes about people living with HIV | *People need to know that even people who are HIV positive, once they are on treatment, their viral load is suppressed and they can get married and they can have babies who are very fine. So, that is something regarding sexual health which I think they are still some misconception and misunderstanding.* |
| Unintentional sexual act | These are the culturally acceptable act which are performed by community members or by parents to their children without knowing that they are sexual acts. | *There are cultures which exist in this country, where we have the elderly when they are socializing with young girls, they have means where one tribe pulls the labia so that they become big, the others pull the clitoris until when it looks like a little penis* |
| Community confusion on sexual issues | These are contradicting information about sexual health from authorities to the community. For example the information they research from their health professional conflicts with the information they head from politicians and other leaders. | *I have a client whom I was counselling for family planning. Then, the client was like you (health professionals) are telling us to use family planning methods, but our president said we should bear kids because education is free* |
| Banning of useful hospital items | This refers to a situation when a government banned the sale of KY gel lubricant by pharmacy. The reason behind was key population are using it against medical prescription. | *For example, a lubricant as K-Y gel, they have to buy from a pharmacy or whatever and once upon [a time] it was forbidden by the ministry.* |
| Homosexuality as a psychological problem | These are believes form some politicians that people who are homosexual have psychological problems and should be given medical or psychological help to become normal or else they should be punished. | *There are people who believe that there are people who are born like that, with those maybe I would say physiological or psychological impairment hence attracted to same sex… or if they have decided to be like this, they need to be punished because this is something which is against our community as well as our laws… But what I know is with a proper education they can become good people with acceptable behaviors. But mind you it needs time to see the impact* |
| Political deception to the society | This is a situation where by politicians avoid speaking about the actual reality of sexual health to the communities especially when they know that the community is against it. | *They [are] pretending that they are against sex workers, but yet sex workers are really few here in Dar es Salaam during parliamentary proceedings in Dodoma (the capital city).* |
| Influences from donors | This refers to use of the projects/items which are funded by donors in health care sectors without checking its quality or side effect, just because government or a scientist is getting money from the funded project/item. | *Yeah, politicians are results of science. Because even if I will go and ask for money for doing research, if insect treated nets are reducing manpower, they won’t give. They will end up saying WHO [the World Health Organization] they have already said that this is safe, [so] who are you by the way? Do you want to reinvent the wheel? WHO, they have already [said] its safe for malaria prevention, man power for what while people are dying for malaria? But again, they are pushed by funders to, and they need that fund [so] they have no option. And when they bring us, we have to take it to the intended are* |
| Perception towards Key Population care providers | These refers to perception of the community toward health care provider who treats KPs friendly. | *The other misconceptions are if society sees you working with key populations (KPs), they consider you as one of the KP also* |
| Servicing Key Population viewed as a promotion | Perception of community members toward anybody who is talking positively about KPs. Most of them they believe that positive viewers are promoting it or they are payed to do so. | *Even within the government, there are people who would view us as puppets, and other say we are fighting for KPs rights because of donors only and they even went further and say we are being paid to promote* |
| Sex topic is a taboo | Perception and believes of church members about sexual health, including talking about it in their jurisdiction. | *We do not say it [talk about it] at church and parents at home also they do not say it* |
| Sex related issues are private | These refers to believers of the churches/Mosques leaders about sexual health talks with their members and if it is possible to talk about it in their jurisdictions. | *People think that this (sex) is something which is so private, very private you cannot speak about it. So sacred, we do not need to talk about it* |
| Sexual deviations as bad characters | These refers to how religious views same sex or other sexual acts apart from penis-vaginal penetration. | *We do not talk about sex of the same sex ... It is an abomination in our context but this is something people are doing and it has effect* |
