# Supplementary material for: Community myths and misconceptions about sexual health in Tanzania: Stakeholders’ views from a qualitative study in Dar es Salaam Tanzania
Source: PLoS One. 2023 Feb 10;18(2):e0264706. doi: 10.1371/journal.pone.0264706 (PMC9916544; doi:10.1371/journal.pone.0264706)
Supplement: S3 File — (DOCX) [file pone.0264706.s004.docx]

**S1 Key stakeholders guide**

--How long have you been in this position (working experience)?

**GENERAL INTRO**: as I said, we are going to talk about sexual health as one of problems which face our country recently? When I’m saying sexual health problems I mean ………………….

**PROBLEM IDENTIFICATION:** In your opinion what are the top sexual health concerns facing Tanzania?

a. Probe: Why do you think these (as mentioned problem) are the top sexual health problems?

b. What are other sexual health concerns do you know/hear from constituents?

1. **UNMET NEEDS AND PRIORITIES**: In looking up at people of your congregation/ community/society background what problems do you encounter that need to be addressed?
   1. Probe: If respondent mentioned any problem, ask him/her, why do you think it’s a problem
2. **PROBLEM IDENTIFICATION:** What is your perception about health professionals when providing sexual health services in their daily practices
   1. ***Probe:*** What do health professionals do/don’t do when working with people?
3. **OPINION:** What is your perception about the current sexual health policy in addressing sexual health needs/problems?
   1. ***Probe:*** Based on the current sexual health policy and sexual reproductive health and rights policy, what worked well and what did not work well in addressing sexual health needs and why?
   2. ***Probe:*** What do you think should be improved in the current/future policy to address the problems mentioned?
   3. ***Probe:*** What do you think should be added in the curriculum to improve the health providers practices?
4. **CURRICULUM**: What should we teach our students about sexual health?
   1. ***Probe***: about relationships between men, women and children you represent?
   2. ***Probe:*** What topics do you recommend we should address and why?
5. **CARE:** To provide sensitive care, what should we teach our students about people based on your experiences/ background?
6. **BUILDING TRUST:** Based on your experience, what are the health professionals’ practices like? explain
   1. ***Probe***: What do you think health professionals do well in treating people based on your experiences?
   2. ***Probe***: What do they do badly in providing health services to clients/patients?
   3. What do you think needs to be done to improve the situation? (PROBLEM IDENTIFICATION and solution)
7. **TABOOS**: Are there any sensitive issues in sexual health care that you have encountered in you daily practices that curriculum designer should be sensitive with? if yes what are they?

**For Political leader**

- 1. ***Probe***: What are the sensitive issues that are discussed/addressed in the parliament session that curriculum designers should be aware of.
  2. ***Probe***: What are the sensitive issues that are discussed outside the parliament session that curriculum designers should be aware of.
  3. ***Probe:*** Please can you give us an example of sensitive issues in sexual health care that draw much attention during parliamentary session and outside the parliament as well.
  4. ***Probe:*** Explore to see if there is any sensitive issues that affect different groups (men, women and children)?

**For Community (NGOs) stakeholders**

1. ***Probe***: What are the sensitive issues that are discussed/addressed in your organization that curriculum designers should be aware of?
2. ***Probe***: What are the sensitive issues that are discussed outside your organization session that curriculum designers should be aware of?
3. ***Probe:*** Explore to see if there is any sensitive issues that affect different groups (men, women and children)?

**For religious leaders**

1. ***Probe***: What are the sensitive issues that are discussed/addressed in your religion that curriculum designers should be aware of?
2. ***Probe***: What are the sensitive issues that are discussed outside your religion session that curriculum designers should be aware of? .
3. ***Probe:*** Explore to see if there is any sensitive issues that affect different groups (men, women and children)?

**For sexual health expert**

1. ***Probe:*** Please can you give us an example of sensitive issues in sexual health care in of your practices
2. ***Probe:*** explore to see if there is any sensitive issue that face different groups (men, women and children)?

**For cultural leaders.**

1. ***Probe***: What are the sensitive issues that are discussed/addressed in your culture that curriculum designers should be aware of?
2. ***Probe***: What are the sensitive issues that are discussed outside your culture that curriculum designers should be aware of? .
3. ***Probe:*** Explore to see if there is any sensitive issues that affect different groups (men, women and children)?
4. **STEREOTYPES**: What are the misconceptions and misunderstandings related to sexual health that you have encountered in your community?
   1. ***Probe:*** Are there any religious, political and cultural labels misunderstandings that are related to sexual health?
5. **AVAILABILITY**: We are thinking of having a panel of expert like yourself present to the students on issues related to sexual health care, would you be interested? *If respondent will not be interested then ask if they can think of someone else who can talk about this to students?*
6. Is there anything else regarding this topic that you want to share with me before ending this interview?
