# Supplementary material for: Community myths and misconceptions about sexual health in Tanzania: Stakeholders’ views from a qualitative study in Dar es Salaam Tanzania
Source: PLoS One. 2023 Feb 10;18(2):e0264706. doi: 10.1371/journal.pone.0264706 (PMC9916544; doi:10.1371/journal.pone.0264706)
Supplement: Attachment — Submitted filename: PONE-D-20-40972_reviewer.pdf [file pone.0264706.s005.pdf]

PLOS ONE

Community Myths and Misconceptions about Sexual Health Promotion in Tanzania: Stakeholders' Views from a Qualitative Study in Dar es Salaam Tanzania

--Manuscript Draft--

Manuscript Number:	PONE-D-20-40972
Article Type:	Research Article
Full Title:	Community Myths and Misconceptions about Sexual Health Promotion in Tanzania: Stakeholders' Views from a Qualitative Study in Dar es Salaam Tanzania
Short Title:	Community Myths and Misconceptions about Sexual Health Promotion in Tanzania
Corresponding Author:	Simon Rosser<br>Univ. MN<br>UNITED STATES
Keywords:	Misconception; misunderstanding; sexual health; reproductive health.
Abstract:	Abstract<br>Introduction: Sexual and reproductive health problems are one of the top five risk factors for disability in the developing world. The rates of sexual health problems in most African countries are overwhelming, which is why HIV and other sexually transmissible infections are still such a challenge in sub-Saharan Africa. Talking about sex in most African countries is a taboo, leading to common myths and misconceptions that ultimately impact community sexual health.<br>Methods: In this study, we conducted 11 key informant individual interviews with community, religious, political, and health leaders in Tanzania for the purpose of understanding common myths and misconception associated with sexual health. Qualitative content analysis was used to analyze all the materials.<br>Results: Two main categories emerged from the analysis. The first category, "Ambiguities about sexual health" focused on societal and political misconceptions and identified ten myths or misconceptions common in Tanzania. Informants highlighted the confusion that happens when different information about sexual health is presented from two different sources (e.g., community leaders/peers and political leaders), which leaves the community and community leaders unsure which one is reliable. The second category, "Practical dilemmas in serving clients", addressed a range of professional and religious dilemmas in addressing sexual health concerns. This included the inability of religious leaders and health care providers to provide appropriate sexual health care because of internal or external influences.<br>Conclusion: Myths and misconceptions surrounding sexual health can prevent communities from adequately addressing sexual health concerns, and make it more difficult for health care providers to comfortably provide sexual health care to patients and communities. Health care providers need to be prepared to challenge these myths and misconceptions, but may have inadequate sexual health training to respond when challenged by community members. An evidence-based comprehensive sexual health curriculum for medical, nursing, and midwifery students is needed to train health professionals with accurate, evidenced based information to treat sexual health concerns.
Order of Authors:	Gift Gadiel Lukumay
	B. R. Simon Rosser
	Lucy R Mgopa
	Stella E. Mushy
	Agnes F Massawe
	Ever Mkonyi
	Inari Mohammed
	Dorkas Mwakawanga
	Maria Trent

Powered by Editorial Manager® and ProduXion Manager® from Aries Systems Corporation

	James Wadley
	Michael W Ross
	Zobeida Bonilla
	Selbada Leshabari
**Additional Information:**	
**Question**	**Response**
**Financial Disclosure**<br><br>Enter a financial disclosure statement that describes the sources of funding for the work included in this submission. Review the submission guidelines for detailed requirements. View published research articles from *PLOS ONE* for specific examples.<br><br>This statement is required for submission and **will appear in the published article** if the submission is accepted. Please make sure it is accurate.<br><br>**Unfunded studies**<br>Enter: *The author(s) received no specific funding for this work.*<br><br>**Funded studies**<br>Enter a statement with the following details:<br>• Initials of the authors who received each award<br>• Grant numbers awarded to each author<br>• The full name of each funder<br>• URL of each funder website<br>• Did the sponsors or funders play any role in the study design, data collection and analysis, decision to publish, or preparation of the manuscript?<br>• **NO** - Include this sentence at the end of your statement: *The funders had no role in study design, data collection and analysis, decision to publish, or preparation of the manuscript.*<br>• **YES** - Specify the role(s) played.<br><br>* typeset	B.R.S.R.<br><br>Study funded by the Eunice Kennedy Shriver National Institute of Child Health and Human Development (NICHD), National Institutes of Health, Grant number: 1 R01 HD092655. The content is solely the responsibility of the authors and does not necessarily represent the official views of the National Institutes of Health.<br><br>The funders had no role in study design, data collection and analysis, decision to publish, or preparation of the manuscript.<br><br>https://www.nichd.nih.gov/
**Competing Interests**<br><br>Use the instructions below to enter a competing interest statement for this submission. On behalf of all authors, disclose any competing interests that	The authors have declared that no competing interests exist

could be perceived to bias this work—acknowledging all financial support and any other relevant financial or non-financial competing interests.<br><br>This statement **will appear in the published article** if the submission is accepted. Please make sure it is accurate. View published research articles from *PLOS ONE* for specific examples.<br><br>**NO authors have competing interests**<br><br>Enter: *The authors have declared that no competing interests exist.*<br><br>**Authors with competing interests**<br><br>Enter competing interest details beginning with this statement:<br><br>*I have read the journal's policy and the authors of this manuscript have the following competing interests: [insert competing interests here]*<br><br>* typeset	
**Ethics Statement**<br><br>Enter an ethics statement for this submission. This statement is required if the study involved:<br><br>• Human participants<br>• Human specimens or tissue<br>• Vertebrate animals or cephalopods<br>• Vertebrate embryos or tissues<br>• Field research<br><br>Write "N/A" if the submission does not require an ethics statement.<br><br>General guidance is provided below. Consult the submission guidelines for detailed instructions. **Make sure that all information entered here is included in the Methods section of the manuscript.**	University of Minnesota institutional review boards (IRB) Case Study: Study00004044, as well as the National Institute for Medical Research in Tanzania.

**Format for specific study types**<br><br>**Human Subject Research (involving human participants and/or tissue)**<br>• Give the name of the institutional review board or ethics committee that approved the study<br>• Include the approval number and/or a statement indicating approval of this research<br>• Indicate the form of consent obtained (written/oral) or the reason that consent was not obtained (e.g. the data were analyzed anonymously)<br><br>**Animal Research (involving vertebrate animals, embryos or tissues)**<br>• Provide the name of the Institutional Animal Care and Use Committee (IACUC) or other relevant ethics board that reviewed the study protocol, and indicate whether they approved this research or granted a formal waiver of ethical approval<br>• Include an approval number if one was obtained<br>• If the study involved *non-human primates*, add *additional details* about animal welfare and steps taken to ameliorate suffering<br>• If anesthesia, euthanasia, or any kind of animal sacrifice is part of the study, include briefly which substances and/or methods were applied<br><br>**Field Research**<br><br>Include the following details if this study involves the collection of plant, animal, or other materials from a natural setting:<br>• Field permit number<br>• Name of the institution or relevant body that granted permission	
**Data Availability**<br><br>Authors are required to make all data underlying the findings described fully available, without restriction, and from the time of publication. PLOS allows rare exceptions to address legal and ethical concerns. See the PLOS Data Policy and FAQ for detailed information.	No - some restrictions will apply

A Data Availability Statement describing where the data can be found is required at submission. Your answers to this question constitute the Data Availability Statement and **will be published in the article**, if accepted.<br><br>**Important:** Stating 'data available on request from the author' is not sufficient. If your data are only available upon request, select 'No' for the first question and explain your exceptional situation in the text box.<br><br>Do the authors confirm that all data underlying the findings described in their manuscript are fully available without restriction?	
**Describe where the data may be found in full sentences. If you are copying our sample text, replace any instances of XXX with the appropriate details.**<br><br>• If the data are **held or will be held in a public repository**, include URLs, accession numbers or DOIs. If this information will only be available after acceptance, indicate this by ticking the box below. For example: *All XXX files are available from the XXX database (accession number(s) XXX, XXX.*).<br>• If the data are all contained **within the manuscript and/or Supporting Information files**, enter the following: *All relevant data are within the manuscript and its Supporting Information files.*<br>• If neither of these applies but you are able to provide **details of access elsewhere**, with or without limitations, please do so. For example:<br><br>*Data cannot be shared publicly because of [XXX]. Data are available from the XXX Institutional Data Access / Ethics Committee (contact via XXX) for researchers who meet the criteria for access to confidential data.*<br><br>*The data underlying the results presented in the study are available from (include the name of the third party*	"This article is based on qualitative interviews with politicians, religious and community leaders, and academics conducted on the condition of anonymity. Because the topics covered are considered very sensitive, even politically dangerous in Tanzania, and potentially could be used against the key informants if someone identified them from the broader transcript, we feel ethically compelled to keep the transcripts private to protect the informant's identities."

and contact information or URL).<br>• This text is appropriate if the data are owned by a third party and authors do not have permission to share the data.<br><br>* typeset	
Additional data availability information:	

Manuscript

Click here to access/download;Manuscript;Misconception - Final.doc

Community Myths and Misconceptions about Sexual Health Promotion in Tanzania: Stakeholders' Views from a Qualitative Study in Dar es Salaam Tanzania.

Gift G. Lukumay MSc.PHEC, BScN[1], Lucy R. Mgopa, MMed, MD[1] , Stella E. Mushy, MN. MWH, BScN[1], B. R. Simon Rosser, PhD, MPH[2], Agnes F. Massae, MN. MWH, BScN[1], Ever Mkonyi, MS, MA[2], Inari Mohammed, MPH[2], Dorkas Mwakawanga, MSc.MWH, BSc.M[1] Maria Trent, MD, MPH[3], James Wadley, PhD[4], Michael W. Ross, PhD, MD DSc[2], Zobeida Bonilla, PhD[2], Sebalda Leshabari, RNM, Ph.D., MPH[1.]

Affiliation:

1. Muhimbili University of Health and Allied Sciences (MUHAS), School of Public Health and Social Sciences, PO Box 65015, Dar es Salaam, Tanzania
2. University of Minnesota, School of Public Health, 1300 S. 2nd St. #300 Minneapolis, MN, USA
3. Johns Hopkins Medicine, 200 N. Wolfe Street, Rubenstein Child Health Building Baltimore, MD 21287 , Washington, DC, USA
4. Lincoln University, School of Adult and Continuing Education, 1570 Baltimore Pike, Lincoln University, PA, USA

Corresponding Author

B.R. Simon Rosser, PhD, MPH, LP
rosser@umn.edu
University of Minnesota
1300 S. 2nd St. #300
Minneapolis, MN 55454
Wk. 612-624-0358; Cell. 612-356-1711; Fax. 612-625-5652

Abstract

Introduction: Sexual and reproductive health problems are one of the top five risk factors for disability in the developing world. The rates of sexual health problems in most African countries are overwhelming, which is why HIV and other sexually transmissible infections are still such a challenge in sub-Saharan Africa. Talking about sex in most African countries is a taboo, leading to common myths and misconceptions that ultimately impact community sexual health.

Methods: In this study, we conducted 11 key informant individual interviews with community, religious, political, and health leaders in Tanzania for the purpose of understanding common myths and misconception associated with sexual health. Qualitative content analysis was used to analyze all the materials.

Results: Two main categories emerged from the analysis. The first category, "Ambiguities about sexual health" focused on societal and political misconceptions and identified ten myths or misconceptions common in Tanzania. Informants highlighted the confusion that happens when different information about sexual health is presented from two different sources (e.g., community leaders/peers and political leaders), which leaves the community and community leaders unsure which one is reliable. The second category, "Practical dilemmas in serving clients", addressed a range of professional and religious dilemmas in addressing sexual health concerns. This included the inability of religious leaders and health care providers to provide appropriate sexual health care because of internal or external influences.

Conclusion: Myths and misconceptions surrounding sexual health can prevent communities from adequately addressing sexual health concerns, and make it more difficult for health care providers to comfortably provide sexual health care to patients and communities. Health care providers need to be prepared to challenge these myths and misconceptions, but may have

inadequate sexual health training to respond when challenged by community members. An evidence-based comprehensive sexual health curriculum for medical, nursing, and midwifery students is needed to train health professionals with accurate, evidenced based information to treat sexual health concerns

Key words: Misconception, misunderstanding, sexual health, and reproductive health.

Introduction

Sexual and Reproductive Health (SRH) is “a state of physical, emotional, mental and social well-being in relation to sexuality, and not merely the absence of disease, dysfunction or infirmity”. [1] SRH is one of the top five risk factors for morbidity in the developing world. [3] The rates of sexual health problems in most African countries are overwhelming, which is why HIV and other sexually transmissible infections (STIs) remain such a challenge in sub-Saharan Africa. [1], [2] In most African countries, talking about sex is taboo and makes many people very uncomfortable. [3] For example, parents believe that it is a teacher’s duty to teach their children about sexual health at school, while teachers think that talking about sex is a basic parental responsibility. As a result, many children end up with no information regarding sexual health. [1], [4] Women and youth are the most frequent victims of sexual violence and suffer a great deal of the sexual health consequences. [1], [4] Across Africa, the reasons why sexual health problems persist are complex, but include lack of sexual health education, taboos against talking about sex, and socio-cultural factors such as living in a male dominated society. [5] The World Health Organization (WHO) has identified gaps in sexual health care and in its current five-year strategic plan, and has prioritized actions for stemming STIs especially in the most affected and vulnerable countries. [6] Across the continent, there are severe consequences from inadequate (or poor) sexual health care, including high rates of infertility, abortion, sexual dysfunction, HIV/ STIs, and long term physical and mental sequelae (e.g., increased rates of certain cancers, depression and neurosyphilis). [7] While these consequences affect all countries, their impact has been observed particularly in some sub-Saharan Africa countries where there are commonly-held myths and misconceptions about sexual health. [3], [4] [8], [9]

In the absence of education, myths and misconceptions are fostered and maintained by community unawareness and incorrect knowledge of sexual health. [8], [9] Health professionals, who the public expects to have a good understanding of sexual health (or at least sufficient knowledge to educate others), may hold the same myths and misconceptions that they grew up with in a particular society. [10–13] As long as health professionals remain within their culture, given the taboos around talking about sex, their beliefs are likely to go unchallenged. Thus, health providers may pass on and reinforce inaccurate information to patients and promote problematic care. Political, religious and cultural factors about sexual health play such a significant role in many African countries that these factors also perpetuate myths and misconceptions in various communities and become resistant to change. [11–13]

Comprehensive sexual health education is an essential component of all health professional's training, and a critical step in ensuring that healthcare professionals and the entire society can confront myths and misconceptions related to sexual health. Educating health professionals will improve both the care patients with sexual health problems receive, as well as the dissemination of accurate sexual health information through education. Well-educated health care providers can confront the negative attitudes, myths and misconceptions related to sexual health found within their communities. However, comprehensive evidence-based sexual health education is not a component of medical, nursing and midwifery curricula in Africa, currently. To address the health concerns of Africa, there is an urgent need to develop sexual health care curricula that will address the sexual health crises impacting our continent. [14]

In 2016, we conducted a 4-day pilot workshop of a sexual health curriculum with nursing and midwifery students at Muhimbili University of Health and Allied Sciences (MUHAS) in Dar es Salaam, Tanzania. This pilot study adapted a PAHO/WHO sexual health curriculum training

for Health Care Providers (HCPs) and tested it in Tanzania. The evaluation showed the sexual health curriculum was acceptable and feasible, [14] but it also identified a need to tailor this curriculum to be African-centric in focus in order to maximize its effectiveness. As part of a formative research phase of study to identify the sexual healthcare needs of Tanzania, we conducted key informant interviews as well as focus groups discussion with midwifery, nursing and medical students and experienced health professionals. In this paper, we report the results from key informant interviews with cultural, religious, political and community (NGO) leaders in Tanzania about common sexual health misconceptions related to sexual health in their jurisdictions. Identifying key myths and misconceptions about sexual healthcare is a critical step in developing an Afro-centric tailored sexual health curriculum. For this study, we define "myth" both as a traditional or widely held but false belief, idea, or practice and "misconception" as a view or opinion that is inaccurate or incorrect based on clinical evidence.

Methods

Study Design

The study used a qualitative exploratory design in which we conducted individual interviews with key stakeholders knowledgeable about the sexual health concerns of their communities and of Tanzanian society.

Data collection and setting

Data were collected from eleven key sexual health stakeholders from June to August 2019 in Dar es Salaam, Tanzania. There were eight male and three female informants. Three were religious leaders (one imam, one priest and one pastor), three experts in sexual health, two

community leaders from non-governmental organizations (NGOs), two cultural experts and one was a politician. Eight of the key interviewees were male, three females, aged 40 to 76 years, with between 8 and 43 years' experience working in their area of specialty.

This study is a collaboration between two universities and was conducted under the oversight of the Muhimbili University of Health and Allied Sciences (MUHAS), the University of Minnesota (UMN), and the National Institute of Medical Research (NIMR), Tanzania.

A purposeful sampling was implemented to identify key informants for the study. The study team identified health, cultural, political and religious leaders who are known to deal with sexual health issues. Key informants were contacted and informed about the study. Those who agreed to participate were interviewed either around the MUHAS campus or at the informant's office (whichever they preferred). All interviews were undertaken in a private room to ensure convenient access and privacy. This allowed the team to capture informants' experience, and identify training needs of current students in each respective discipline.

Key informant interviews were chosen as an effective method to explore, in depth, the interviewee's information, perceptions and experiences of the topics under study. Each interview, lasted between 45 to 60 minutes. The interviews were conducted by bilingual interviewers in English or Kiswahili or a mix of both languages. At the start of the interview, the informant was invited to respond in whichever language they preferred, with the majority preferring to use English.

Before commencing the interview, each informant completed a short demographic form and reviewed the consent forms. The interviewer then highlighted that the interview was voluntary, that the interviewee could discontinue at any time, that all responses would be kept

confidential (meaning not attributed to them personally), and that the session was being audiotaped.

The interview guide consisted of nine questions that addressed three broad areas: The first area was about sexual health problem identification, unmet sexual health needs, and the impact of sexual health policies in Tanzania. The semi-structured interview was formulated to cover multiple areas, and tailored to the expert's area of expertise. Each key informant was asked to identify the top sexual health concerns facing Tanzania, unmet needs and priorities, their opinion on how well health providers provide sexual health services, the impact of policy in addressing sexual health problems, what a sexual health curriculum for health students should cover, identification of myths, and misconceptions and stereotypes about sexual health in their communities. This paper reports the experts' responses to the questions about common myths and misconceptions.

Data management and analysis

After each interview, audio files were labeled with the interview ID and uploaded onto a secure server. The audio files were transcribed verbatim and then translated into English (where necessary). In addition, notes taken during the interview were typed on a shared password protected drive.

Coding approach. A deductive-inductive coding strategy informed by grounded theory principles was employed to develop the codebook and code the transcripts. A team-based approach to coding and codebook development was implemented, [15], [16] open coding, first coding cycle, axial coding. This approach to data analysis enabled a process of debate and

reflection that generate a richer understanding of the stakeholder's experiences in serving their community, in relation to sexual health.

Coding team. The coding and data analysis team was composed of health professionals currently practicing in their respective fields and conducting research. These providers contributed their experiences as practitioners and researchers to the team enriching the data collection and data analysis process, and serving as important member checks during the development of code definitions and data interpretations.

Codebook. Codebook development was an iterative process that was initiated with the creation of a list of deductive codes derived from the interview guide. Both deductive and inductive codes continued to be generated and added to the codebook iteratively during the three phases of coding. This was accomplished via regular team meetings and the use of a Google Doc where coders engaged in a process of continuous written feedback and updates to the codes and definitions that led to reconciliation of disagreements and refinement of the codebook.

Open coding/discovery of themes. Open coding was conducted during a regular meeting with the team of interviewers. Open coding involved reading, several times, three hard copy transcripts of the interviews and coding the interviews manually. During this meeting, the team identified early ideas and emerging themes using an open-ended coding format, without predetermined codes or categories. Pens, color highlighters, sticky notes, and large flip charts were used during this stage to gather the early ideas and organize the codes under emerging themes. Teams organized in groups of two coders and specific questions were assigned to the

team based on expertise, background, and interests. Each group manually coded the transcripts individually and compared notes and codes at the end of the open coding stage. Teams had an 80% or higher initial agreement. Codes generated during the open coding were entered into the main codebook.

Team members who conducted the interview continued with the coding. Each team coded questions pertaining to these areas of expertise and interests. All responses to questions in those topics were assigned to each sub team. Each team coded between 2-3 questions across all interviews. Each sub team met regularly to consult on the meaning of codes, reconcile differences, reach agreements, and updates to the codebook on the team document housed on the shared drive.

First coding cycle. During the first coding cycle, the team coded all transcripts. A holistic/segmentation process was followed to code the transcripts. In this coding process, meaning was ascribed to responses by each question contained in the interview.

Axial coding. During axial coding, each sub team generated larger categories based on the findings of the first coding cycle. Codes were grouped accordingly under these larger findings

Results

All stakeholders described a strong commitment to working with the community, making sure that the people they serve understand them and vice versa. Stakeholders spoke of feeling discouraged when the community does not follow rules, regulations, or their advice. But they also reported that they find courage, and calm themselves in order to fulfil their duties of serving

their particular community. Informants identified an especially challenging situation is when the community does not understand or “speak” the same “expert” language, increasing the risk of the community having misconceptions and misunderstandings of what you’re telling them.

Informants stated it was critical to address the common myths and misconceptions about sexual health in the communities they serve. From our analysis of the responses of stakeholders, two categories emerged: (1) *Ambiguities about sexual health* and (2) *Practical dilemmas in serving clients*. This first refers to both the actual myths and misconceptions that arise, and when different information comes from two different sources (e.g., community leaders/peers and politicians at different times). This leaves the community and community leaders unsure which one is reliable. *Practical dilemmas in serving clients* refers to the inability of leaders (religious, cultural and health care) to provide appropriate sexual health care because of internal or external influences (see Table 1).

Category 1: Ambiguities about Sexual Health

Participants pointed out that in addition to their primary role of informing the community on many things including sexual health issues, they also have to confront some widely held myths and misconceptions. While these can come from anywhere, informants referred often to misconceptions and misunderstandings coming from political leaders. These widely shared myths and misconceptions are passed down from generation to generation, or they persist because uninformed and/or misinformed people are resistant to change. Some informants went further to express concern that while politicians are educated, they can use or even promote community misunderstanding for their own political benefit. Informants described ambiguities as stemming from two main categories: societal and professional misconceptions.

Societal Misconceptions

Key informants described different communities as having specific misconceptions related to sexual health (see Table 2). These myths and misunderstandings were based on inadequate knowledge in a particular community or society. One interviewee explained that in some communities, people have wrong perceptions about family planning methods and vaccine usage. When health professionals encourage people to use family planning or to get a vaccine, the recipients tend to refuse. Alternatively, they fear that the family planning methods or a vaccine will be harmful to them. Some may articulate that family planning or vaccines are a white racist conspiracy to weaken Africa.

"They think family planning is not of good intention. They say they (white people) want to decrease the size of the population in sub-Saharan [Africa]." (Non-Government Organization Representative 2)

Similarly, the distribution of human papilloma vaccine to adolescent school girls in Tanzania is not effective because parents decline to consent, fearing that these vaccines will cause infertility in their daughters and because they do not believe the vaccine will prevent cervical cancer.

"As usual in some of African countries, you find parents are refusing to give consent and say that there is some hidden agenda behind the vaccine. They don't believe that it is there just to protect their children from cancer… Well, they say, that we (the vaccine providers) want to stop

their girls from getting children, we are sterilizing them." (Non-Government Organization Representative 2)

However, in some cases women are confused with the information they receive from the health professionals and political leaders regarding sexual health, because the information given may be contradictory to one another and therefore the recipients are left with so many uncertainties.

"I have a client whom I was counseling for family planning. Then, the client was like you (health professionals) are telling us to use family planning methods, but our president said we should bear kids because education is free." (Non-Government Organization Representative 1)

While there is an assumption that sex is voluntary, informants said that this is not always true in some African cultures. A sexual act may be misinterpreted and may carry different meanings within a particular society. For instance, some traditional healers would require either their patients or young boys and girls to have sex with them as a means of treating an illness.

"*If you go to a traditional healer and [he] tells you must go have sex with him or a young girl or a young boy, for you that's tricky. For me, that is sex and a ritual … parents to play around with the penis of the young boys, and then they say, ahaaa, and the boy would laugh, in fact and that...that...when I now think about it that was sex socialization." (Cultural expert 2)*

In other African cultures, in order for a parent to ascertain his son's manhood or sexual arousal, a child's parent may rub his son's penis and observe the young boy's reaction. This is considered normal in some African cultures.

"*These are cultures which exist in this country, where we have the elderly when they are socializing with young girls, they have means where one tribe pulls the labia so that they become big, the others pull the clitoris until when it looks like a little penis" (Cultural expert 2).*

Given the taboo of talking about sex, and at the family level, the absence of parental sex education, it is difficult for young people to know what is right or wrong. As one informant explained, a man may rub himself against a woman's behind to the point of ejaculation in a congested public place like in a bus or at the market without her being aware. Without education that this is not normal, and that frotteurism is diagnostic of a paraphilia, the youth may end up either with others minimizing his behavior or being taken into custody instead of being referred for assessment and treatment.

"For example, when you talk about parent-youth communication, when they want to talk about sexual health related issues, so this norm of not talking is a misconception … It is really difficult for a father [in Tanzanian culture] to start talk with his child daughter about sex, so it is a part of that challenge." (Health Expert 2)

A public health expert emphasized misconceptions regarding HIV patients. Some community members maintain a misconception that once someone is infected with HIV they cannot get

married to anyone or bear children because they will infect the partner or the baby. In this era of antiretroviral therapy (ARV), women who are on successful on ARV treatment can be married and have children without infecting their partner.

"People need to know that even people who are HIV positive, once they are on treatment, their viral load is suppressed and they can get married and they can have babies who are very fine. So, that is something regarding sexual health which I think they are still some misconception and misunderstanding." (Health Expert 3)

Political Misconceptions

Politics does have its own contribution towards sexual health misconceptions, which affects the community. Stakeholders described a tendency where politicians use community misunderstandings, or the reserved culture, to advance their agenda. Participants pointed out that there are occasions where political representatives contribute to community misunderstanding towards HIV prevention. For example, the Tanzanian government banned the sale of K-Y gel lubricant by pharmacies. The stated reason behind the ban was key populations (i.e., men who have sex with men) use lubricant. The politician's stated concern was that the lubricant violates the law by facilitating same sex relations which are illegal. But informants noted that lubricants can also be used by heterosexuals, women may need them for gynecological exams, lubricant reduces friction during sexual intercourse for menopausal women, and lowers the risk of HIV transmission.

"For example, a lubricant as K-Y gel, they have to buy from a pharmacy or whatever and once upon [a time] it was forbidden by the ministry. Is that not a misunderstanding that we want to stop transmission of HIV and then you, as a boss, you stop using this, and this is one of the tools that one has to use to minimize the friction, sometimes the rate of transmission of HIV." (Health Expert 2).

Informants stated that many politicians avoid speaking about the actual reality of sexual health to the communities. Most politicians were perceived as pretending to be against key populations such as sex workers or men who have sex with men and overstate their contribution to the HIV epidemic in the country caused by having multiple sex partners, while minimizing heterosexual risk behaviors.

"They [are] pretending that they are against sex workers, but yet sex workers are really few here in Dar es Salaam during parliamentary proceedings in Dodoma (the capital city)." (Cultural Expert 2)

Informants said some politicians in Tanzania use the excuse that sex education might influence innocent youth to practice such behaviors. While politicians are supposed to be role models, they deny or stigmatize some behaviors while engaging in them privately. Informants saw this as a deception to the community because it makes the politician look good while depriving the community of receiving important information regarding sexual health.

"*So, with the politicians, … many of them will oppose the very behaviors they practice, and particularly because the practices are private and no one will know. But when they are in public, that is the language they speak which is different." (Cultural expert 2)*

Some politicians advocate that people who are homosexual have psychological problems and should be given medical or psychological help to become normal (i.e., heterosexual), while others advocate that since they choose to practice same sex relations, then they should be punished.

" *There are people who believe that there are people who are born like that, with those maybe I would say physiological or psychological impairment hence attracted to same sex… or if they have decided to be like this, they need to be punished because this is something which is against our community as well as our laws… But what I know is with a proper education they can become good people with acceptable behaviors. But mind you it needs time to see the impact."(Political leader)*

Category 2: Practical Dilemmas

Participants reported some practical challenges and dilemmas in addressing sexual health concerns in Tanzania. Informants noted that because politicians have public power, they can announce misinformation regarding sexual health. Regardless of the truth of what they assert, it becomes difficult to challenge the public statement which has already been accepted by the people. Cultural experts claimed that most of the sexual health services in Tanzania are donor oriented. Therefore, they are fund driven and difficult to query their effectiveness. Religious

leaders saw themselves as committed to preaching the word of God primarily and not about advocating for sexual health (regardless of how much they know about it). There were two types of practical dilemmas informants encountered: professional misconception and religious-based misconceptions.

Professional Misconception

Participants reported that conflicts emerge when professionals claim one reality when the community members experience another. For example, in family planning, the professionals are vulnerable to believing the science that a medication tested elsewhere in the world is fine, while the community members are reporting side effects. This can lead to the professionals minimizing the community's experience.

"But, here, the science will say there are no problems but users had seen those problems, so for you coming from MUHAS (i.e., the Health Sciences University), what you hear from these individuals those are misconceptions. But these are reality in their own world and you're the one who have misconceptions on what they are saying." (Cultural Expert 1)

In less developed countries like Tanzania, most of the health services are donor funded. When members of community report problems with the inefficiency of a particular service or the quality of the item donated to them (e.g., mosquito nets), the health professionals may deny, minimize or disregard the concern raised by the community and condemn them for expressing concern. Instead, health professionals support the donors who supply the items and the funds because of the risk of destroying the political or professional relationships, and ultimately, of

losing the funding. Health professionals must be accountable, however, to research and confirm the communities' concerns to better engage sponsors about the needs of the community.

"*Yeah, politicians are results of science. Because even if I will go and ask for money for doing research, if insect treated nets are reducing manpower, they won't give. They will end up saying WHO [the World Health Organization] they have already said that this is safe, [so] who are you by the way? Do you want to reinvent the wheel? WHO, they have already [said] its safe for malaria prevention, man power for what while people are dying for malaria? But again, they are pushed by funders to, and they need that fund [so] they have no option. And when they bring us, we have to take it to the intended area." (Cultural Expert 1)*

Furthermore, when dealing with high risk populations, the community and health professionals may have significant misconceptions towards sexual health care in relation to key populations (i.e. sex workers, men who have sex with men, prisoners, and individual with substance use disorders). There is a stigma for health care providers who treat sex workers or gay men that they practice same sexual behaviors or are promoting the behavior as if guilty by association. This leads to discrimination and segregation from other care providers based on their personal moral values. Likewise, if a political leader supports human rights and equal health care services to all regardless of profession, sex orientation, or risk behavior, they may be misperceived as an activist by the community and fellow politicians rather than a professional who is promoting quality care for all to ensure the health of the overall population.

"The other misconceptions are if society sees you working with key populations (KPs), they consider you as one of the KP also. So, even in health care facilities, other health workers they start to segregate you… Even within the government, there are people who would view us as puppets, and other say we are fighting for KPs rights because of donors only and they even went further and say we are being paid to promote" …(Political leader)

Religious misconceptions

Religious leaders also identified barriers and misconceptions regarding sexual health in those they serve. Regardless of the religion or denomination, they said discussion about sexual health topics in churches or mosques was considered shameful and taboo. Religious informants considered sex a private matter while acknowledging the importance of talking about it. Many sexual health issues were considered unmentionable because of the belief that sex is a sacred act reserved for marriage. This view was not only held by the religious key informants but also, in their experience, by their congregations. In addition, behaviors like same sex relationships and anal sex were highly regarded as immoral behaviors which were against their religious values.

"People think that this (sex) is something which is so private, very private you cannot speak about it. So sacred, we do not need to talk about it … We do not say it [talk about it] at church and parents at home also they do not say it… We do not talk about sex of the same sex ... It is an abomination in our context but this is something people are doing and it has effects." (Religious leader 2)

Participants acknowledged that many sexual practices and health concerns are common in our society, but people do not talk about them, hence no help is provided to those with the concern. They acknowledged a role for religious leaders in confronting the silence around sexual health concerns.

"I have encountered some cases whereby there was a religious school and people are complaining that kids are introduced issues related to anal sex by either staff, or other peers from suburbs. You find children are congested together in rooms…. So, we need to talk about these issues. Because nobody who is exempted when you are talking about sex, these staffs cover everybody and we need to help." (Religious leader 3)

Discussion

This study identified ten common myths and misconceptions viewed by stakeholders related to sexual health in Tanzania (see Table 2). These findings provide insight into what sexual health myths and misconceptions stakeholders encounter when serving their communities. These can be used to identify critical knowledge gaps that need to be addressed when developing a sexual health curriculum in Tanzania. Our findings indicate that there are several common myths and misconceptions that are prevalent among community members across different jurisdictions.

Myths and misconceptions which were raised by respondents included negative perceptions on family planning, mistrust of health care providers who treat key populations, vaccines especially the effects of the HPV on fertility, infectivity of people living with HIV, whether traditional practices are healthy or abusive, and whether homosexuality and anal sex

should be rejected or treated. Across their varying expertise, all the experts identified the cultural taboo about talking about sexual issues/topics as the major overarching issue sustaining myths and misconceptions.

With regards to family planning, experts said community members perceived greater health risks of using modern family planning methods than the evidence suggests, believing that they are more harmful than as described in educational leaflets or based on health care providers' advice.[4], [9], [17], [18] Community members may often believe that there is a hidden agenda to promote infertility, population control, and weight gain. This is despite the documented health benefits associated with use of modern contraceptives.[19], [20] These misconceptions leave women of reproductive age without effective methods of pregnancy prevention and increase the risk of unplanned pregnancies.

The benefits of HPV vaccination for cervical cancer and anogenital wart prevention has been documented scientifically and it has been proved safe for use. [3], [21] Key informants stated that community members perceive HPV vaccination as a strategy to depopulate their community by sterilizing adolescent girls. Low uptake of HPV vaccination will ultimately result in needlessly high rates of cervical cancer and other anogenital conditions.[3] In addition, these perspectives lead to mistrust between care providers and their clients due the introduction of suspicion into the patient-provider relationship. This finding is consistent with studies in other countries that have documented the association between such myths and misconceptions about the safety of the HPV vaccine and suboptimal vaccination rates.[10], [22]

Additionally, in this era of effective antiretroviral (ARV) treatment, the viral load of individuals living with HIV can be suppressed to the point where HIV may not be transmitted during pregnancy, child birth, or sexual intercourse.[7], [23] Despite this, community members

were described as continuing to have negative perceptions toward people living with HIV and exaggerated risk of HIV transmission to partners and children even with effective treatment. Similar misconceptions have been reported in other countries.[6], [24]–[26] Such misconceptions may infringe on the legal rights of individuals living with HIV to marry and parent.

Misconceptions about and stigmatization of key populations (especially men who have sex with men) was common, severe and pervasive.[27]–[29] Informants identified other health care providers as stigmatizing colleagues who provide care to key population patients. High quality, evidence-based care was reframed as promoting same sex sexual behavior or advocacy. Health care workers who provide high quality care, regardless of patient background were viewed with suspicion. These findings are consistent with research conducted in Jamaica and the Bahamas.[30] All health care workers take an oath to provide treatment without prejudice, therefore, the inter-collegial suspicion and stigmatization noted by key informants in this study is especially concerning. This toxic and unprofessional behavior perpetuates suboptimal care, may lower morale in the healthcare workforce.

In addition to the usual sex health challenges that exist in all or almost all countries, key informants raised the challenge of ritual sexual practices in some tribes and cultures. This includes sex as a treatment between traditional healers and patients, and also genital touching between older relatives and children. Traditional healers' sexual practices, especially intercourse with patients, is both an HIV/STI concern, and a form of sexual exploitation.[31]

Myths and misconceptions are a major reason community member do not follow medical advice across several areas.[5], [10], [24], [32] This was thought to emerge when community leaders promote information that is counter to evidence-based medical advice. This result in

confusion within the community as to what information to believe. Several informants described scenarios in which health care providers have advised the community based on scientific evidence,[14], [33] but this information contradicts views of some politicians. Politicians were often described as deliberately promoting misinformation in some cases to advance their careers. Hypocrisy also emerged as an issue given that some politicians publicly denouncing some sexual behaviors as immoral while privately engaging in them.[17]

Lastly, churches, masjids, and other houses of worship where the community gathers are potential places to promote accurate sexual education, positive attitudes, and open discussion. However, religious leaders confirmed that they consider sex as a private matter that should not be spoken about publicly. This perspective was present despite leaders knowing that their members have or may be at risk of a number of sexual problems. There was also a fear expressed by some informants that talking about a sexual issue may encourage others, especially the young, to experiment. In religious groups the taboo/culture of not talking about sex.[11]–[13] prevents open discussion of sexual health and maintains sexual misconceptions.

In many countries, there are misconceptions regarding sexual health but they may be exacerbated in low-income countries.[4] Lack of education in general, and lack of access to accurate sexual information in particular, likely maintain all of the myths and misconceptions identified by the key informants. Encountering providers who hold these beliefs or are unprepared to counteract them may deter patients who have sexual health problems from seeking care. Without high quality sexual health advice and care, patients may engage in sexual risk-taking behaviors that result in poor health outcomes including high rates of unwanted pregnancies, HIV/STI, sexual exploitation and abuse. To dispel these myths and misconceptions, it is important to provide comprehensive evidence-based sexual health and sexuality education

for health professionals. In the Tanzanian context, our results indicate that sexual health education should specifically address the effectiveness and benefits of family planning, HPV vaccination, ARV treatment, and non-judgmental practices of providers towards clients and colleagues who serve key populations at risk for sexual health disparities.

In most high-income countries, medical, nursing and midwifery students have access to sexual health education as part of their professional training,[34] which is not the case in most African countries. To be effective in Africa, such a curriculum needs to address the issues identified by the key informants and potentially other unidentified areas to ensure a comprehensive education for clinical practice. Students and practicing professionals need training so that they can learn accurate information about sexual behavior and clinical health issues, how to address sexual health problems using evidence-based interventions, and how to provide sexual education and services within the community. Once such a health curriculum is evaluated, broad dissemination to include professional training programs and continuing education should be a priority.

We expect that after health care providers are trained in sexual health, they will begin to educate their patients during clinic visits and through community education. Social support, and Train the Trainer programs among the community workers will be a potential adjunct to create broad knowledge within the community given that myths and misconceptions have a negative impact to individual and community health. [35]–[38]

Limitations of the study

In this study, we interviewed diverse key stakeholders and community leaders trying to understand common misconceptions and misunderstanding related to sexual health in different

communities in Tanzania. Eleven interviews were conducted for the present study, which might seem a small sample. However, during the last three interviews, the same issues emerged repeatedly suggesting saturation was being reached. While the interviews were conducted in both Swahili and English, the scripts were translated to and analyzed in English. We acknowledge that some of the meaning of the participants might have been lost in the translation process. To minimize this, six of the authors, all bilingual in English and Kiswahili were involved in the analysis.

Conclusions and recommendations

This study has identified several key sexual health myths and misconceptions that function as a barrier to clients and the community seeking sexual health care. These myths and misconceptions also create a barrier to health care providers providing quality sexual health care, especially stigmatized and vulnerable patient groups. Health care providers need to be prepared to challenge these myths and misconceptions, but may have inadequate sexual health training to respond when challenged by community members. An evidence-based comprehensive sexual health curriculum for medical, nursing, and midwifery students is needed to train health professionals with accurate, evidenced based information to treat sexual health concerns. This curriculum in Tanzania should specifically address the common myths and misconceptions identified by the key informants in this paper and provide opportunities to practice key communication skills for optimal clinical effectiveness and community advocacy.

Table 1: Category, Sub-category and Codes Which Emerged During Analysis.

Category	subcategory	Selected Codes
Ambiguities about Sexual Health	Society Misconception	Negatives perceptions on Family Planning and vaccines<br>Sex as a ritual<br>Mislabeling of HIV patients<br>Unintentional sexual act<br>Community confusion on sexual issues
	Political misconceptions	Banning of useful hospital items<br>Homosexuality as a psychological problem<br>Political deception to the society
Practical dilemma on serving client	Professional misconception	Influences from donors<br>Perception towards Key Population care providers<br>Servicing Key Population viewed as a promotion
	Religions misconceptions	Sex topic is a taboo<br>Sex related issues are private<br>Sexual deviations as bad characters

Table 2. Common Community Myths and Misconceptions about Sexual Health in Tanzania, as reported by Key Informants	
**Myth or Misconception**	**Scientific or Clinical View**
1. Family planning is an attempt by white people to decrease the size of the population in Sub-Saharan Africa	1. Access to modern contraception expands education opportunities for women, lowers infant mortality, and at the national level, sustains population growth and economic development [39]
2. The human papilloma vaccine causes infertility and makes girls fat and ugly.	2. There is no causal link between HPV vaccine and infertility. A large US-based study found young girls with obesity were less likely to have been HPV vaccine than normal weight girls [40]
3. Family planning is against some religions, and national interest	3. Tanzania has one of the highest rates of teen pregnancy, unplanned pregnancy in the word. An estimated 13.4% pregnancies in TZ are unplanned and unwanted [41]
4. Traditional healers need to have sex with young patients for the treatment to be effective	4. In western societies, this would be viewed as sexual abuse and is illegal. [42]
5. In some tribal cultures, parents and elders play with a boy's penis to socialize the boy sexually.	5. In western societies, this would be viewed as sexual abuse and is illegal. [42]
6. In some tribal cultures, parents and elders pull the labia and/or clitoris of young girls to make	6. In western societies, this would be viewed as sexual abuse and is illegal. Labial elongation is an effective minimal risk technique [43]

them grow and to socialize the girl sexually	
7. Educating particularly boys about sex will make them want to have sex.	A review of 87 studies found sex education did not lead to earlier sexual activity. A third of programs delayed sexual debut, decreased frequency of sex, and number of sexual partners [44]
8. People who are HIV positive should not have sex or children in case they transmit the virus.	8. HIV positive persons with undetectable viral load cannot transmit HIV including to sex partners, and have <1% transmission during pregnancy, labor and delivery [45]
9. Homosexuality is a disorder that can be treated	9. Homosexuality was removed as a diagnostic disorder in the DSM in 1973 and form the ICD-10 in 1990. There are no evidence-based effective treatments to change a person’s sexual orientation [46]
10. Banning lubricant e.g. K-Y gel will stop men from having sex with men.	10.Lubricant e.g. KY gel, lowers risk of HIV/STI transmission by decreasing the risk of condoms breaking. [47] There is no evidence that banning lubricant deters men from having sex with men or stops anyone from anal sex.

References

[1] Singh RK, Patra S. What Factors are Responsible for Higher Prevalence of HIV Infection among Urban Women than Rural Women in Tanzania? *Ethiop J Health Sci*. 2015 25(4):321-328. doi:10.4314/ejhs.v25i4.5.

[2] World Health Organization, *2018 Report on global sexually transmitted infection surveillance*. 2018.

[3] Rothman SM, Rothman DJ. Marketing HPV vaccine: implications for adolescent health and medical professionalism. *JAMA*. 2009 302(7):781-786. doi:10.1001/jama.2009.1179

[4] Lee J, Jezewski MA. Attitudes toward oral contraceptive use among women of reproductive age: a systematic review. *ANS Adv Nurs Sci*. 2007 30(1):E85-E103. doi:10.1097/00012272-200701000-00016

[5] Tay SK, Tesalona KC, Rashid NM, Tai EY, Najib SM. Vaccine Misconceptions and Low HPV Vaccination Take-up Rates in Singapore. *Asian Pac J Cancer Prev*. 2015 16(12):5119-5124. doi:10.7314/apjcp.2015.16.12.5119

[6] Sano Y, Antabe R, Atuoye KN, Hussey LK, Bayne J, Galaa SZ, et al. Persistent misconceptions about HIV transmission among males and females in Malawi. *BMC Int Health Hum Rights*. 2016 Jun 7;16(1):16. doi: 10.1186/s12914-016-0089-8. PMCID: PMC4896031.

[7] Kilewo C, Karlsson K, Ngarina M, Massawe A, Lyamuya E, Swai A, et al. Prevention of mother-to-child transmission of HIV-1 through breastfeeding by treating mothers with triple antiretroviral therapy in Dar es Salaam, Tanzania: the Mitra Plus study. *J Acquir*

Immune Defic Syndr. 2009 Nov 1;52(3):406-16. doi: 10.1097/QAI.0b013e3181b323ff. PMID: 19730269. [Online]. Available: www.jaids.com.

[8] Tyler CP, Whiteman MK, Zapata LB, Curtis KM, Hillis SD, Marchbanks PA. Health care provider attitudes and practices related to intrauterine devices for nulliparous women. *Obstet Gynecol*. 2012 Apr;119(4):762-71. doi: 10.1097/AOG.0b013e31824aca39. PMID: 22433340.

[9] Gueye A, Speizer IS, Corroon M, Okigbo CC. Belief in Family Planning Myths at the Individual and Community Levels and Modern Contraceptive Use in Urban Africa. *Int Perspect Sex Reprod Health*. 2015 41(4):191-199. doi:10.1363/4119115

[10] Albright AE, Allen RS. HPV Misconceptions Among College Students: The Role of Health Literacy. *J Community Health*. 2018 Dec;43(6):1192-1200. doi: 10.1007/s10900-018-0539-4. PMID: 29922992.

[11] Metusela C, Ussher J, Perz J, Hawkey A, Morrow M, Narchal R, et al. "In My Culture, We Don't Know Anything About That": Sexual and Reproductive Health of Migrant and Refugee Women. *Int J Behav Med*. 2017 Dec;24(6):836-845. doi: 10.1007/s12529-017-9662-3. PMID: 28620774.

[12] Shaw SJ, Huebner C, Armin J, Orzech K, Vivian J. The role of culture in health literacy and chronic disease screening and management [published correction appears in *J Immigr Minor Health.* 2009 Dec;11(6):531. Orzech, Katherine [corrected to Orzech, Kathryn]]. *J Immigr Minor Health*. 2009 11(6):460-467. doi:10.1007/s10903-008-9135-5

[13] Sterling SP, Sadler LS. Contraceptive use among adolescent Latinas living in the United States: the impact of culture and acculturation. *J Pediatr Health Care*. 2009 23(1):19-28. doi:10.1016/j.pedhc.2008.02.004

[14] Ross MW, Leshabari S, Rosser BRS, Trent M, Mgopa L, Wadley J, et al. Evaluation of an assessment instrument for a sexual health curriculum for nurses and midwifery students in Tanzania: The sexual health education for professionals scale (SHEPS). *Appl Nurs Res*. 2018 Apr;40:152-156. doi: 10.1016/j.apnr.2018.01.005. Epub 2018 Feb 1. PMID: 29579491.

[15] Saldaña, J. "The Coding Manual for Qualitative Researchers." [Online]. Available: www.sagepublications.com. 2015 Nov 2

[16] McLellan E, MacQueen KM, Neidig JL. Beyond the qualitative interview: Data preparation and transcription. *Field methods*. 2003 Feb;15(1):63-84., doi: 10.1177/1525822X02239573.

[17] Hamani Y, Sciaki-Tamir Y, Deri-Hasid R, Miller-Pogrund T, Milwidsky A, Haimov-Kochman R. Misconceptions about oral contraception pills among adolescents and physicians. *Hum Reprod*. 2007 22(12):3078-3083. doi:10.1093/humrep/dem259.

[18] Mbachu CO, Agu IC, Obayi C, Eze I, Ezumah N, Onwujekwe O. Beliefs and misconceptions about contraception and condom use among adolescents in south-east Nigeria. doi: https://doi.org/10.21203/rs.2.18742/v2

[19] World Health Organization, "Reducing unsafe abortion from unintended pregnancies," 2014. [Online]. Available: http://www.guttmacher.org/.

[20] Chola L, McGee S, Tugendhaft A, Buchmann E, Hofman K. Scaling Up Family Planning to Reduce Maternal and Child Mortality: The Potential Costs and Benefits of Modern Contraceptive Use in South Africa. *PLoS One*. 2015 10(6):e0130077. Published 2015 Jun 15. doi:10.1371/journal.pone.0130077

[21] Taira AV, Neukermans CP, Sanders GD. Evaluating human papillomavirus vaccination programs. *Emerg Infect Dis*. 2004 10(11):1915-1923. doi:10.3201/eid1011.040222.

[22] Tay SK, Tesalona KC, Rashid NM, Tai EY, Najib SM. Vaccine Misconceptions and Low HPV Vaccination Take-up Rates in Singapore. *Asian Pac J Cancer Prev*. 2015 16(12):5119-5124. doi:10.7314/apjcp.2015.16.12.5119

[23] Townsend CL, Cortina-Borja M, Peckham CS, de Ruiter A, Lyall H, Tookey PA. Low rates of mother-to-child transmission of HIV following effective pregnancy interventions in the United Kingdom and Ireland, 2000-2006. *AIDS*. 2008 22(8):973-981. doi:10.1097/QAD.0b013e3282f9b67a

[24] Ekstrand ML, Bharat S, Ramakrishna J, Heylen E. Blame, symbolic stigma and HIV misconceptions are associated with support for coercive measures in urban India. *AIDS Behav*. 2012 16(3):700-710. doi:10.1007/s10461-011-9888-z

[25] Bogart LM, Skinner D, Weinhardt LS, Glasman L, Sitzler C, Toefy Y, et al. HIV misconceptions associated with condom use among black South Africans: an exploratory study. Afr J AIDS Res. 2011 Jan 1;10(2):181-187. doi: 10.2989/16085906.2011.593384. PMCID: PMC3144581.

[26] Fraim NL. Knowledge levels and misconceptions about HIV/AIDS: what do university students in Turkey really know. *Int J Humanit* Soc Sci. 2012 2(2):50-8. [Online]. Available: https://www.researchgate.net/publication/265905400.

[27] Fitzgerald-Husek A, Van Wert MJ, Ewing WF, Grosso AL, Holland CE, Katterl R, et al. Measuring stigma affecting sex workers (SW) and men who have sex with men (MSM): A systematic review. *PLoS One*. 2017 Nov 30;12(11):e0188393. doi: 10.1371/journal.pone.0188393. PMCID: PMC5708696.

[28] Stahlman S, Hargreaves JR, Sprague L, Stangl AL, Baral SD. Measuring Sexual Behavior Stigma to Inform Effective HIV Prevention and Treatment Programs for Key Populations. *JMIR Public Health Surveill*. 2017;3(2):e23. Published 2017 Apr 26. doi:10.2196/publichealth.7334.

[29] Lyons CE, Ketende S, Diouf D, Drame FM, Liestman B, Coly K, et al. Potential Impact of Integrated Stigma Mitigation Interventions in Improving HIV/AIDS Service Delivery and Uptake for Key Populations in Senegal. *J Acquir Immune Defic Syndr*. 2017 Jan 1;74 Suppl 1(Suppl 1):S52-S59. doi: 10.1097/QAI.0000000000001209. PMCID: PMC5147043.

[30] Rogers SJ, Tureski K, Cushnie A, Brown A, Bailey A, Palmer Q. Layered stigma among health-care and social service providers toward key affected populations in Jamaica and The Bahamas. *AIDS Care*. 2014 26(5):538-546. doi:10.1080/09540121.2013.844762

[31] Bove R, Valeggia C. Polygyny and women's health in sub-Saharan Africa. *Soc Sci Med*. 2009 68(1):21-29. doi:10.1016/j.socscimed.2008.09.045

[32] Bal MD, Özkan SA. Misconceptions about family planning of women in Turkey. *J Hum Sci*. 2015 Apr 21;12(1):1319-29.

[33] Parish SJ, Clayton AH. Sexual medicine education: review and commentary. *J Sex Med*. 2007 4(2):259-268. doi:10.1111/j.1743-6109.2007.00430.x.

[34] Schutt-Aine J, Maddaleno M. Sexual health and development of adolescents and youth in the Americas: program and policy implications. Washington, DC: Pan American Health Organization; 2003 Feb.

[35] Vuylsteke B, Semdé G, Sika L, Crucitti T, Ettiègne Traoré V, Buvé A, et al. HIV and STI prevalence among female sex workers in Côte d'Ivoire: why targeted prevention programs

should be continued and strengthened. *PLoS One*. 2012;7(3):e32627. doi: 10.1371/journal.pone.0032627. Epub 2012 Mar 5. PMCID: PMC3293836.

[36] Khan MS, Unemo M, Zaman S, Lundborg CS. HIV, STI prevalence and risk behaviours among women selling sex in Lahore, Pakistan. *BMC Infect Dis*. 2011;11:119. Published 2011 May 11. doi:10.1186/1471-2334-11-119

[37] Muraguri N, Tun W, Okal J, Broz D, Raymond HF, Kellogg T, et al. HIV and STI prevalence and risk factors among male sex workers and other men who have sex with men in Nairobi, Kenya. *J Acquir Immune Defic Syndr*. 2015 Jan 1;68(1):91-6. doi: 10.1097/QAI.0000000000000368. PMCID: PMC4973514.

[38] Desai VK, Kosambiya JK, Thakor HG, Umrigar DD, Khandwala BR, Bhuyan KK. Prevalence of sexually transmitted infections and performance of STI syndromes against aetiological diagnosis, in female sex workers of red light area in Surat, India. *Sex Transm Infect*. 2003 79(2):111-115. doi:10.1136/sti.79.2.111

[39] World Health Organization, *Family planning: a global handbook for providers. 2018*.

[40] Harris JA, Garrett AA, Akers AY. Obesity and Disparities in Human Papillomavirus Vaccination for U.S. Adolescent Girls and Young Women. *Womens Health Issues*. 2019;29(1):31-37. doi:10.1016/j.whi.2018.09.007

[41] Exavery A, Kanté AM, Njozi M, Tani K, Doctor HV, Hingora A, et al. Predictors of mistimed, and unwanted pregnancies among women of childbearing age in Rufiji, Kilombero, and Ulanga districts of Tanzania. *Reprod Health*. 2014 Aug 8;11:63. doi: 10.1186/1742-4755-11-63. PMCID: PMC4130430.

[42] Shaw TA, Herkov MJ, Greer RA. Examination of treatment completion and predicted outcome among incarcerated sex offenders. *Bull Am Acad Psychiatry Law*. 1995;23(1):35-41.

[43] Martínez Pérez G, Namulondo H. Elongation of labia minora in Uganda: including Baganda men in a risk reduction education programme. *Cult Health Sex*. 2011 Jan;13(1):45-57. doi: 10.1080/13691058.2010.518772. PMID: 20960354..

[44] Paris, "BOOK REVIEWS United Nations Educational, Scientific and Cultural Organization International Technical Guidance on Sexuality Education: An evidence-informed approach for schools, teachers and health educators," 2009.

[45] Fauci AS, Redfield RR, Sigounas G, Weahkee MD, Giroir BP. Ending the HIV Epidemic: A Plan for the United States. *JAMA*. 2019;321(9):844-845. doi:10.1001/jama.2019.1343

[46] Drescher J. Out of DSM: Depathologizing Homosexuality. *Behav Sci (Basel)*. 2015;5(4):565-575. Published 2015 Dec 4. doi:10.3390/bs5040565

[47] Sullivan PS, Carballo-Diéguez A, Coates T, Goodreau SM, McGowan I, Sanders EJ, et al. Successes and challenges of HIV prevention in men who have sex with men. *Lancet*. 2012 Jul 28;380(9839):388-99. doi: 10.1016/S0140-6736(12)60955-6. Epub 2012 Jul 20. Erratum in: Lancet. 2012 Jul 28;380(9839):340. PMCID: PMC3670988.
