# Supplementary material for: Community myths and misconceptions about sexual health in Tanzania: Stakeholders’ views from a qualitative study in Dar es Salaam Tanzania
Source: PLoS One. 2023 Feb 10;18(2):e0264706. doi: 10.1371/journal.pone.0264706 (PMC9916544; doi:10.1371/journal.pone.0264706)
Supplement: Attachment — Submitted filename: Comment to authors.pdf [file pone.0264706.s007.pdf]

Further comments to authors

I thank the authors for addressing my previous comments. I am pleased with the revision but have one more observation, particularly on data analysis section.

In line 187-192: “Coding approach”. The authors state “An inductive coding strategy informed by grounded theory principles was employed to develop the codebook and code the transcripts.” Which implies that only an inductive approach was engaged in developing codes. Yet, in line 201-203, the author state “Both deductive and inductive codes continued to be generated and added to the codebook iteratively during the three phases of coding.” Did the analysis only engage the inductive approach? If so, the statement in lines 201-203 is irrelevant. In case the analysis engaged both inductive and deductive strategies in developing codes and coding the data, it is important that in lines 187-192 where the authors talk about the coding approach, they also mention the deductive approach in addition to the inductive one. For example, a statement like, “The inductive and deductive strategies were employed to develop the codebook and code the transcripts. First, a series of inductive codes was developed based on the principles of Grounded Theory. Second, the deductive coding was performed based on xxx.”.

Good luck!
