# Supplementary material for: Community myths and misconceptions about sexual health in Tanzania: Stakeholders’ views from a qualitative study in Dar es Salaam Tanzania
Source: PLoS One. 2023 Feb 10;18(2):e0264706. doi: 10.1371/journal.pone.0264706 (PMC9916544; doi:10.1371/journal.pone.0264706)
Supplement: Attachment — Submitted filename: Response to Reviewers-Gift 26-12-2021 SR edits.doc [file pone.0264706.s006.doc]

**MUHIMBILI UNIVERSITY OF HEALTH AND ALLIED SCIENCES**

**SCHOOL OF NURSING-COMMUNITY HEALTH NURSING DEPARTMENT**

P.O. Box 65004

DAR ES SALAAM

TANZANIA

Tel G/Line: +255-22-2150302/6 Ext. 1213

Direct Line: +255-22-2152467

Telefax: +255-22-2150465

E-Mail: giftgadiel@yahoo.com

**22^nd^ Dec 2021**

To: Managing Editor

Plos One

**RE: REVIEWERS RESPONSES.**

Dear Editor and Reviewers:

Thank you for your helpful comments and positive review of our manuscript entitled “Community Myths and Misconceptions about sexual health in Tanzania: Stakeholders' Views from qualitative study in Dar es Salaam Tanzania”. Below please find a point-by-point summary of the comments (in regular type) together with our response (in italics).

**Reviewer 1**

I'd like to thank the authors for attempting to address my and reveiwer2's previous comments. I am happy with this revision but have further suggestions...

Abstract:
the authors mentioned in their response letter that they opted for "stakeholders" but there's informants in line 34.

*We apologize. We have replaced the synonyms (informants) with “stakeholders” as suggested by the reviewer throughout the paper.*

Introduction
Please check your referencing... only one box is needed to house the ref number... e.g., myths and misconceptions are common in Tanzania [11-15] and not [11] - [15], no, this is not how it goes... This comment applies throughout the manuscript in text referencing. If the references are not chronological you can still put them in one square box this way [5, 7,11-4] ... Hope this is making sense. Watch out for spacing before the in text refencing/ square boxes.

*Thank you for this reminder. We confirm we have checked the referencing and have placed only one box to house the reference number/s as required.*
Methods
line 125, please rearrange to read: Sampling and data collection process

*We agree, we have rearranged the subheading to read “Sampling and Data Collection Process” as advised by the reviewer.*

line 139, here's key informants again...? I expected to see the reference to the "Additional file" of the interview guide in this section but I cannot find it...It would be ideally places somewhere by line 148

*Thank you for reminding us again, as described early we have replaced the other synonyms(informants) with “stakeholders” as suggested by the reviewer throughout the paper. And we have attached the interview guide as recommended at first mention of the guide.*

Nicely described analysis process!

*Thank you for your kind comment.*

Results
Table 2, page 14... there's a typo- .... unplanned pregnancies in the "world"
line 277, another "informant" and in line 327, 338, 348... there's more, please ensure you check this for consistency!

*Thank you. We have corrected the typo, and replaced the word “informants” with “stakeholder” throughout the paper for consistency.*

Otherwise, this is a nice paper and timely for health providers.

*Thank you again for your kind assessment and appreciation*

Reviewer 3: 
Thanks to Plos One for the opportunity to review this manuscript and to the authors for their interesting work on this important topic. Generally, the paper is well-written. The authors did a good job in addressing the comments raised in a previous round of review. However, there are some issues in the manuscript that need to be addressed before it can be considered for publication. I have outlined these concerns below and hope that my comments are helpful to the authors if they are given the opportunity to revise the paper:


Introduction
-The introduction is fairly written. But I wonder if in Tanzania there are no studies that addressed myths and misconceptions around sexual health. I would urge the authors to acknowledge the existing evidence and situate their study within it.

*Thank you for your nice comment. Initially, in the introduction, we had only two references directly from Tanzania [References 1 and 14]. We have added several more. Reference number 2 is from WHO in which Tanzania is involved too; reference number 4 is a systematic review from many countries including from Tanzania. And we have added other studies from Tanzania to enrich the study [see reference numbers 10-12]. These now appear on Page 5.*

-It would be good to add some lines stating the situation of sexual health problems in Tanzania and the related myths and misconceptions.

*Thank you for the comment. A paragraph about situation of sexual health in Tanzania and related myths and misconception has been added on Page 5*.

Methods
-Lines 131-133, the authors state: “The study team identified health, cultural, political and religious leaders who were known to deal with sexual health issues”. It is not clearly described who helped the researchers in identifying and approaching these key informants to participate in the interviews.

*Participants of these study were selected purposively, the authors who live and work in Tanzania, together with our colleagues form the US met in January 2019 to identify who would be the most appropriate stakeholders to interview. Our dear late Dr. Leshabari who was co-Principal Investigator and senior faculty at MUHAS was the primary decision maker. All the stakeholders selected by our team are public figures known to deal with sexual health publicly via several media. The process is clearly described in last paragraph of page 7.*

-Under the sub-section “Setting”, line 145-146, the authors state: “Data were collected from June to August 2019 in Dar es Salaam, Tanzania.” I think the authors should provide further description of the setting of this study. This is particularly important for qualitative studies as it provides a context which would be helpful for the reader to understand and interpret the findings of this study. It would also be useful to specify the reason as to why Tanzania, and Dar es Salaam in particular was considered suitable for this study among other others.

*Thank you for this comment. We have described the reason why Dar es salaam is a study area for this study. Please see page 7.*

-Line 145 “setting” please rephrase this to “study setting”

*Thank you, we have rephrased it to “Study Setting.”*

-In lines 146—148 (page 8) the authors state: “For each interview, the team decided in advance who would be most appropriate person to interview each stakeholder, matching wherever possible, the interviewer and stakeholder by gender, age, and other key characteristics.” In the methods section, would be good to specify the age of the interviewers involved in collecting data for this study, and clarify the potential effects of the interviewers’ age on the data collection.

*Age range of clinical faculty who conducted the interview ranged from 35 to 60 years*. See page 8

The potential effects of the interviewers’ age on the data collection

*This allows stakeholders(interviewee) and interviewer to be more confrontable during the interview and share what they know related to sexual health.* See page 8

-Lines 125-134: It is important for the authors to clarify in the “data collection and sampling” section about what guided the decisions on the sample size engaged in generation of data for this study. Was it guided by the principle of saturation? If so, what were the steps used in determining if the saturation was achieved? According to the authors, the prior actual number of participants in this study was anticipated to be 2-3 experts in each category, and indeed they interviewed 2 cultural leaders, 3 sexual experts, 2-community leaders, and 3 religious’ leaders. Can authors comment about this coincidence? Additionally, given the diversity of the categories of participants (sexual health experts, religious, community, and political leaders) engaged in this study, it would be useful if the authors could clarify if the saturation was reached for each type of participants. I can see some highlights on data saturation in the section about the limitation of the study (lines 560-561). I would encourage you to move this information earlier in the sampling sub-section.

*We confirm our study team included a qualitative expert methodologist who trained all members in qualitative methods including the goal of saturation. Our goal was to have 2-3 experts in each category participate so as to attain overall saturation, and indeed we had 2 cultural leaders, 3 sexual experts, 2-community leaders, and 3 religious’ leaders in which we reached saturation from the information we got from them. The exception was politicians. We tried to recruit multiple politicians but were only able to recruit one who was willing to discuss sexual health and key populations. So, in that category, we were unable to confirm if saturation as reached or not. This information is now included as a limitation in the limitations section. We have also included this information in the methodology section. Please see page 9.*

-Lines 137-138: “This allowed the team to capture stakeholders’ experiences, and to identify the training needs of current students in each respective discipline.” Which students? I though the data reported in this paper are based on the stakeholders’ narratives on myths and conceptions about sexual health. Please clarify.

*It’s important to understand that the information obtained from this study had two objectives. As researchers, we wanted to document the common myths and misconceptions related to sexual health in Tanzania. More immediately, we wanted to identify community myths to tailor a sexual heath curriculum for health students in Tanzania. We confirm we have integrated the findings from this study into the curriculum which is currently being evaluated.*

-Lines 143-144: The author state: “The interviewers were clinical faculty in medicine and midwifery from MUHAS…”. This sentence is confusing. Please check.

*All interviewers were faculty members (lecturers) from Muhimbili University of Health and Allied Sciences. Two of them were midwives teaching in the School of Nursing, and one was a medical doctor teaching in the School of Medicine. As clinical faculty, they all are also clinicians working at the Muhimbili National hospital.*

-Line 145: “All interviewers were bilingual in English and Kiswahili.” Please remove this information as it is already mentioned in lines 142-143.

*Thank you for observation. We have removed the repeated line as suggested by reviewer. Please see page 8.*

-Lines 145-146: “...were female…” –Missing punctations (replace the word “female” with “females”)

*Thank you for observation. We have corrected that.*

-In lines 149-150, the authors state: “At the start of the interview, the stakeholder was invited to respond in whichever language they preferred”. Do you mean any language or you wanted to mean that the participants were given freedom to choose between Swahili and English languages? Please check and rectify.

*Again, thank you for observation. We have changed the sentence to read “At the start of the interview, the stakeholder was invited to respond in either English or Swahili, with the most preferring to use English.”*

-In the analysis section (Lines 168-169), the authors state: “A deductive-inductive coding strategy informed by grounded theory principles was employed to develop the codebook and code the transcripts.” As the author may be aware, unlike the inductive coding approach, the deductive coding is not informed by the grounded theory principles. Please correct that statement.

*Thank you for the reminder. We have corrected that statement. Please see page 9.*

-Lines 189-190: “This paper reports the responses to the questions about common myths and misconceptions” Please add “about sexual health”.

*Thank you. We have added the word about sexual health, now the sentence read. “This paper reports the responses to the questions about common myths and misconceptions about sexual health”*

-Lines 189-190: “Open coding involved reading several times three hard copy transcripts of the interviews and coding the interviews manually.” Given the diversity of the participants (i.e., religious, health experts etc.), I wonder if the tree transcripts reviewed for open coding were representative of the sub-populations involved in this study.

*Thank you for an observation. We apologize for the confusion. We read one transcript several time for each subpopulation. Therefore, we read four transcripts and coded them manually in this initial phase to determine early ideas and emerging themes by using an open-ended coding format. We have also deleted the word three and put four in revised manuscript for clarity. Please see page 10.*

-Strongly advise that you include the codebook as a supporting material.

*Thank you, the codebook has been added as a supporting material as suggested by the reviewer.*

-Lines 562-563: “While the interviews were conducted in both Swahili and English, the scripts were translated to and analyzed in English.” It is important to also include this information in the data analysis section.

*We agree. This detail now appears in line 218 of the Data Management and Analysis section.*

Results
Lines 287-293: The information mentioned here is about other contexts in Africa. This make me wonder whether the study explored about the myths and misconceptions around sexual health specific to Tanzania only or any other setting that that the participants knew about?

*We were clear with stakeholders that we were talking about common myths and misconceptions in Tanzania. In their responses, some stakeholders identified a myth or misconception which they said was not limited to Tanzania only, but also was common in other African countries. That is why we presented it in that way.*

-Lines 411-423: “…First, both the community and health professionals may hold significant misgivings about…of the overall population.” Is the information presented in these lines informed by the study findings? The way it is presented now sounds like the authors’ assumptions.

*We confirm it is informed by the study’s findings. Specifically, stakeholders referenced that the community is concerned that health care providers who provide services to key populations (i.e., men who have sex with men, sex workers) are, in fact, promoting or endorsing such behavior.*

Discussion
-The discussion section is generally good, but engagement with a broader range of qualitative literature in Tanzania around perceptions, beliefs and myths around sexual health would help. Put simply, how did the study findings align with other studies in Tanzania?

*We have added a paragraph relating to findings of this study with similar findings from prior research in Tanzania.*

-The policy implications of the findings are not clear.

*We agree. As we hope we have made clear, our aim in this study was to inform and tailor a sexual health training curriculum for health students in Dar es Salaam. It was not to inform policy.*

-I have concerns about some of the conclusions drawn from the manuscript, particularly the suggestion in lines 549-550 where the authors state: “We expect that after health care providers are trained in sexual health, they will begin to educate their patients during clinic visits and through community education”. Surely the authors—as they described in the introduction section—know that the cultural and social context informs health workers’ behaviors/practices, including those related to sexuality. For instance, it has been shown that in Tanzania (Mbekenga et al., 2013 <https://doi.org/10.1186/1472-698X-13-4>, Mchome et al., 2020 <https://doi.org/10.1111/mcn.13048>), despite representing the medical discourse, health care workers emerged as conveyors of the myths and misconceptions around sexuality and breastfeeding during postpartum period. These evidences suggest that awareness or knowledge messages will hardly shift strongly held cultural norms around sexuality. Thus, efforts more than education are needed to make health workers willing and courageous to talk about sexual issues / topics with their clients.

*We respectfully agree in part and disagree in part.*

*Here’s where we agree. In our focus groups with 121 providers and healthcare students (not reported here), we heard several accounts where, in the absence of sexual health education and sexual counseling skills development, medical doctors, nurses and midwives held similar myths and misconceptions around sexual health as the community. For example, they worried that providing a sexually active 14-year-old girl with contraception could encourage sexual promiscuity, that treating HIV or STIs in a man who has sex with men might encourage homosexuality, or that reporting a man for raping or beating his wife might have negative effects on the marriage. We note our findings are consistent with Mbekenga et al.’s observation that “health care workers were sometimes described as conveyors.” But Mbekenga et al. emphasizes “sometimes,” and we also appreciate their caveat that “public health services were not discussed much in the FGDs.” We note the Mchome et al. (2020) study was conducted in a single rural village in Kilosa where there is no health facility, and no doctors, nurses or midwives. Instead, the study interviewed birth attendants and community health workers who are less trained and therefore possibly more likely to convey myths and misconceptions.*

*Where we disagree is in the conclusion that awareness or knowledge will hardly shift strong held cultural norms around sexuality. We respectfully would suggest that it is too sweeping a generalization. And it is not based in evidence. In both the pilot we conducted and the randomized controlled trial of the tailored sexual health curriculum currently in progress at MUHAS, we are seeing large shifts in the knowledge and attitudes of medical, nursing and midwifery students who received sexual health education compared to the waitlist control group (data still under analysis). This suggests that a strong curriculum can significantly modify health students’ attitudes, beliefs, and counseling skills in Tanzania. It may not be perfect, but our students are strongly committed to practicing evidence-informed healthcare. It seems, if you provide students in Tanzanian with sexual health education, many/most will modify their beliefs accordingly.*

-I would suggest adding directions for future research at the end of the discussion so that others interested in this topic of research know how to use this manuscript in the future.

*We agree and have added a paragraph on this.*

Thank you to the reviewers for their helpful feedback.

On behalf of the authors,

Best regards,

*Gift Lukumay, BScN, MSc.PHEC*

Department of Community Health Nursing

Muhimbili University of Health and Allied Sciences
